# Protective effect of a hydroethanolic extract from *Bowdichia virgilioides* on muscular damage and oxidative stress caused by strenuous resistance training in rats

**DOI:** 10.1186/s12970-014-0058-3

**Published:** 2014-12-24

**Authors:** Jymmys Lopes dos Santos, Rafaela Eugênia Arce Dantas, Clésio Andrade Lima, Silvan Silva de Araújo, Elis Cristiane Valença de Almeida, Anderson Carlos Marçal, Charles dos Santos Estevam

**Affiliations:** Postgraduate program in Physical Education, Department of Physical Education, Federal University of Sergipe, São Cristóvão, SE 49100-000 Brazil; Federal University of Sergipe, Cidade Universitária Prof. José Aloísio de Campos, Department of Morphology, Av. Marechal Rondon s/n, Jardim Rosa Elze, São Cristóvão, Sergipe 49100-000 Brazil; Laboratory of Natural Product Chemistry and Biochemistry, Department of Physiology, Federal University of Sergipe, São Cristóvão, SE 49100-000 Brazil

**Keywords:** Oxidative stress, Physical exercise, Hydroethanolic extract

## Abstract

**Background:**

Natural antioxidants can reduce oxidative damage caused by high-intensity resistance training (RT). We investigated the in vitro antioxidant potential of hydroethanolic extract (HEE) from *Bowdichia virgilioides* on muscular damage and oxidative stress in rats subjected to high-intensity RT.

**Methods:**

Thirty-two male Wistar rats were divided into four experimental groups: 1) control group (CG), oral administration (P.O.) of vehicle; 2) trained group (TG), vehicle-treated with RT; 3) *B. virgilioides* untrained group (BVG), treated with *B. virgilioides* HEE (200 mg/kg P.O.); and 4) trained *B. virgilioides* group (TBVG), treated with *B. virgiliodes* HEE (200 mg/kg P.O.). All animals were habituated to the training apparatus for 1 week. CT and TBVG animals were subjected to the training protocol, which consisted of three sets of 10 repetitions with 75% of the load established using the one-repetition maximum, for four weeks. CG and BVG animals were manipulated and fixed to the apparatus three times a week with no load. Treatment with *B. virgilioides* HEE or vehicle treatment was initiated after 25 days of RT (5 days; one dose per day). At the end of the experiments, plasmatic and gastrocnemius samples from all groups were obtained for the assessment of lipid peroxidation and creatine kinase activity.

**Results:**

Compared to TG rats, TBVG rats showed decreases in plasma and gastrocnemius tissue lipid peroxidation by 55.68% (p <0.0001) and 66.61% (p <0.0012), respectively. Further, compared to TG rats TBVG rats showed decreases in plasma and gastrocnemius tissue oxidative stress by 62.83% (p <0.0005) and 54.97% (p <0.0197), respectively.

**Conclusions:**

*B. virgilioides* HEE treatment reduced markers of oxidative stress caused by high-intensity RT. Further, HEE treatment during training significantly reduced the markers of tissue damage.

## Background

Physical exercise is characterized as any form of activity that induces a series of physiological responses in the body and maintains physical fitness [1-3]. Resistance training (RT) is defined as physical activity involving voluntary contractions of skeletal muscles against an external resistance or force that opposes motion, such as one’s own body mass, free weights and other equipment, or manual resistance machines [2,3].

RT is considered a safe form of exercise and is practiced by individuals of various age groups. Further its health benefits are well recognized [4,5], including increased muscular resistance and force [6] and reduced blood sugar levels in diabetic individuals [7]. However, during high-intensity RT, muscles undergo periods of ischemia-reperfusion [8], resulting in increased reactive oxygen species (ROS) levels, including hydroxyl (HO•), alkoxy (RO•), peroxyl (ROO•), and superoxide (O2•-) radicals, and non-radicals such as hydrogen peroxide (H_2_O_2_), singlet oxygen (^1^O_2_), and ozone (O_3_) [9,10]. These molecules can react with proteins, lipids, carbohydrates, and nucleic acids, leading to changes in cell function and cell death. Moreover, ROS are associated with post-exercise inflammatory responses, which can propagate muscular damage [11].

Oxidative stress is a detrimental condition characterized by an imbalance of oxidants and antioxidants [12,13]. It can be caused by overtraining, xenobiotics, exposure to pollutants, use of antibiotics, and UV radiation [14]. Further, high-intensity RT can cause microtears in muscular tissue. Leukocytes and other immune cells migrate to the site of tears, thereby triggering increased ROS production and activating inflammatory mediators [15]. Moreover, Hawke [16] and Saxton *et al*. [17]*.* stated that muscular damage and inflammation are proportional to exercise intensity. These injuries may be related to both the contractile and non-contractile muscle components, such as the extracellular matrix, sarcolemma, and basal membrane [18-21]. However, other studies suggest that chronic exercise may cause depletion of antioxidants, which may increase exercise-induced oxidative stress and tissue damage if combined with diminished ingestion of antioxidants [22].

Numerous approaches have been developed to prevent or minimize the deleterious effects of oxidative stress, including the use of natural and synthetic antioxidants, such as vitamin C (ascorbic acid), E (α-tocopherol), A (β-carotene), and polyphenols from medicinal plants [9,23,24]. Moreover, recent studies suggest that foods rich in polyphenols can reduce oxidative damage in response to physical exertion caused by high-intensity RT [25-27]. Diminished lipid peroxidation and DNA damage was observed in rodents that received supplementation with grape seed oil extract, which contains a high concentration of polyphenols [28-30]. These data suggest that supplementation with antioxidants might reduce oxidative stress and thereby attenuate muscular damage after high-intensity exercise.

In this study, we investigated the in vitro antioxidant potential and protective effects of the hydroethanolic extract (HEE) of *Bowdichia virgilioides* on muscular damage in rats subjected to high-intensity RT. We hypothesized that supplementation with *B. virgilioides* can reduce lipid peroxidation and prevent muscle injury in rats undergoing high-intensity RT.

## Methods

### Animals and treatment period

Thirty-two male Wistar rats (3 months old, weight: 200–250 g) were obtained from the bioterium at Federal University of Sergipe. The rats were randomly housed (four rats per cages) and maintained in temperature-controlled conditions (22 ± 3°C) with a light–dark cycle of 12 h (lights on between 0600 h and 1800 h), free access to food (Labina®), and water ad libitum. All procedures described in this study were approved by the Animal Research Ethics Committee at Federal University of Sergipe (protocol 10/12).

**Figure 1:**
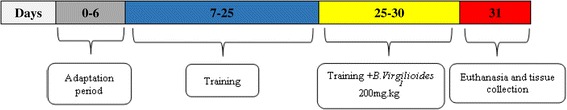
**Organogram of the experimental protocol.** The experiment was performed over 30 days with all animal groups: Day 0 – 6 were the adaptation period without charge (white bars); day 7–25 of training without the use of *B. virgilioides*, day 26 – 30 (5 days with intake of the extract *B. virgilioides*) after initiating RT. At day 31 the animals were euthanized.

The animals were divided into four groups: 1) control group (CG, n = 8), composed of healthy, vehicle-treated animals (Tween 80, 3% P.O., Vetec, LTDA, Rio de Janeiro, Brazil) receiving electrostimulation; 2) trained group (TG, n = 8), composed of healthy vehicle-treated animals (Tween 80, 3% P.O.) subjected to the RT protocol; 3) *B. virgilioides* group (BVG, n = 8), composed of healthy animals treated with *B. virgilioides* HEE (200 mg/kg, P.O); and 4) trained and *B. virgilioides* treated group (TBVG, n = 8), composed of animals subjected to RT and treated with *B. virgilioides* HEE (200 mg/kg, P.O). All animals were either vehicle-treated or received *B. virgilioides* HEE on day 25 of the RT protocol (5 days total treatment, as described in the training protocol), which is shown in the organogram (Figure 1).

The inner bark of *B. virgilioides* was collected in March 2011 from the village of Fazenda Riachão, in the municipality of Japaratuba, Sergipe, Brazil (10°32′04.49 S, 36°53′57″ W). A reference sample of this species was stored in the herbarium at the Federal University of Sergipe under the reference ASE 23.107.

### High-performance liquid chromatography

The high-performance liquid chromatography (HPLC) system used includes a Shimadzu Prominence chromatograph composed of two LC-6 AD pumps, an autoinjector, DGU 20 A5 degasser, a solvent selector valve, and a photodiode detector (DAD SPD M20A). For chromatographic analysis, two C18 columns were used, as well as an analytical column (25.0 × 0.46 cm, 5 mm particles) and a preparatory column (25.0 × 2 cm, 5 mm particles), both manufactured by Shimadzu. To obtain and process the data, we used the chromatographic software LC Solution.

### Antioxidant potential and redox properties of hydroethanolic extract of *B. virgilioides*

HEE samples were dissolved in methanol to obtain a stock solution of 0.5 mg/mL, from which aliquots were removed and added to a solution of 2,2-difenil-1-picrilhidrazina (DPPH•, 40 μg/mL, Sigma-Aldrich, Steinheim, Germany) to obtain a final concentrations of 5, 15, and 25 μg/mL in a reaction volume of 3 mL. The blank was composed of a mixture of the analyzed sample and methanol (Vetec, LTDA, Rio de Janeiro, Brazil). Gallic acid (Abiquim, São Paulo, Brazil) was used as the positive control.

The absorbance value of each sample was obtained using an spectrophotometer at a wavelength of 515 nm, and the readings were taken at 1, 5, and 10 min, and at 10 min intervals thereafter, up to 60 min [31]. The percentage of DPPH remaining (DPPH_REM_%) was calculated according to previous methods [32] using the following equation:

$$ \mathrm{D}\mathrm{P}\mathrm{P}{\mathrm{H}}_{\mathrm{REM}}\% = \left(\mathrm{DPPH}\right)\ \mathrm{T}\ /\ \left(\mathrm{DPPH}\right)\ {\mathrm{T}}_0\times 100 $$

where [DPPH] T is the concentration of radicals in the reaction medium after reaction with the sample; and [DPPH] T_0_ is the initial concentration of DPPH. From the DPPHREM% values, the percent inhibition at 60 min was calculated.

### Measuring lipid peroxidation in vitro

The capacity of HEE to inhibit lipid peroxidation was determined by monitoring the production of thiobarbituric acid reactive substances (TBARS) in the lipid medium, according to previous methods [32]. For the quantification of TBARS, we used the protocol from Lapenna *et al.* [33]. Briefly, 1.0 mL of egg yolk homogenate (1% w/v) was completely dissolved in 20 mM phosphate buffer solution (pH 7.4), and then homogenized with 0.1 mL of HEE at varying concentrations (50, 100, and 200 μg/mL) suspended in methanol.

Lipid peroxidation was induced upon the addition of 0.1 mL of 0.17 M 2,2′-azobis(2-amidinopropane) dihydrochloride (AAPH, Sigma-Aldrich, Steinheim, Germany) and 0.17 M solution of iron sulfate (FeSO_4_, Sigma-Aldrich, Steinheim, Germany) at different time points. Trolox (Sigma-Aldrich, Steinheim-Germany) was used as the positive control, and the extract and solvent (water or methanol) were used as the negative control. The reactions were incubated 30 min at 37°C. After cooling, the samples (0.5 mL) were centrifuged in the presence of 0.5 mL of 15% trichloroacetic acid (TCA, Vetec, LTDA, Rio de Janeiro, Brazil) at 1,200 *g* for 10 min. A 0.5-mL aliquot of the supernatant was mixed with 0.5 mL of 0.67% thiobarbituric acid (TBA, Sigma-Aldrich, Steinheim, Germany) and heated at 95°C for 60 min. After cooling, the absorbance was measured using a spectrophotometer at a wavelength of 532 nm. The results were expressed as percent inhibition.

### Training protocol

RT was carried out using a squat machine. The animals wore a leather jacket, connected to a mobile 35 cm long wooden bar, and the loads were allocated. The rats wearing jackets remained sitting down with their back legs bent and supported, according to the model by Tamaki *et al*. [34]. All animals underwent habituation to the apparatus for one week, where they received electrostimulation. After this period, the CT and TBVG animals were subjected to the training protocol in three sets of 10 repetitions, with rest intervals of 60 s, at an intensity of 75% of the load established using the one-repetition maximum (1RM) test. The RT was performed three times a week on alternate days, for four weeks [35]. The training load and intensity were adjusted every two weeks following a new 1RM test. The CG and BVG animals were manipulated and fixed to the apparatus three times a week on alternate days with electrostimulation, by using three sets of 10 repetitions and a rest interval of 60 s. These animals experienced no load, 0% intensity (Table 1).

**Table 1:** **Resistance training protocol**

**Week**	**Intensity (%)**	Days of the week*	**Sets**	**Repetitions**	**Interval (s)**
**1st**	75	3	3	10	60
**2nd**	75	3	3	10	60
**3rd**	75	3	3	10	60
**4th**	75	3	3	10	60

*Alternate days. The training was conducted for 4 weeks on alternate days at 75% intensity defined by MRI with 3 sets and 10 repetitions with 60-s intervals between a series and another.

Electrical stimulation was applied to animals during each set (20 V/0.3 s in duration, 3 s interval) using electrodes (ValuTrode, Model CF3200, Axelgaard, Fallbrook, CA, USA) fixed to the tail and connected to an electrostimulator (BIOSET, Physiotonus Four, Model 3050, Rio Claro, SP, Brazil).

### Collection of biological material

Twenty-four hours after the last session, the animals were fasted overnight, anesthetized using sodium thiopental (40 mg/kg, i.p., Cristália, Itapira São Paulo, Brazil) and sacrificed. Blood was collected by cardiac puncture, and the rats were decapitated. After the blood was collected, it was immediately centrifuged at 800 *g* for 15 min at 4°C. The supernatant was then stored at −80°C. The organs were removed, and the gastrocnemius muscle was washed three times in a solution of 1.15% KCl (Vetec, LTDA, Rio de Janeiro, Brazil), dried, and weighed. The muscle was then homogenized, and each gram of tissue was mixed with 5 mL of KCl, 10 μL of phenylmethylsulfonyl fluoride (PMSF, 100 mmol, Sigma-Aldrich, Steinheim, Germany), and 15 μL of 10% Triton. The homogenate was then centrifuged at 3,000 *g* for 10 min at 4°C. The supernatant was stored at −70°C until further analyses of oxidative stress and tissue damage markers.

### Biochemical analysis

The biological materials (plasmatic fraction) were analyzed for markers of tissue damage and oxidative stress according to the methodology described by Branco *et al*. [36]. The quantification of tissue damage caused by high-intensity RT was assessed by measuring enzyme markers of tissue damage, such as creatine kinase (CK), lactate dehydrogenase (LDH), alanine aminotransferase (ALT), and aspartate aminotransferase (AST). For quantification, a commercial kit (Labtest®, Santa Lagoa, Minas Gerais, Brazil) was used. Plasma (20 μL) from each animal was homogenized in specific reagents at 37 ± 0.2°C, and readings were taken using a spectrophotometer (Bioespectro Model SP-22 UV/Visible, Minas Gerais, Brazil) at a wavelength of 340 nm.

To determine lipid peroxidation, TBARS was measured according to Lapenna *et al*. [33]. For the assessment of carbonyl proteins, the oxidation of proteins was assessed by determining carbonyl residues (CR) according to the methodology of Faure and Lafond [37].

### Statistical analyses

The results are presented as the mean ± standard deviation (SD). Differences between samples were considered statistically significant when *p* <0.05. All the analyses were carried out in triplicate. After assessing the normality of the data using the Shapiro Wilk test, the data were statistically analyzed using one-way analysis of variance (ANOVA), followed by the Bonferroni or Dunnett multiple comparison tests, when appropriate. The statistical software Graph Pad Prism version 5.0 was used.

## Results

### Antioxidant potential and redox properties of *B. virgilioides* HEE

**Figure 2:**
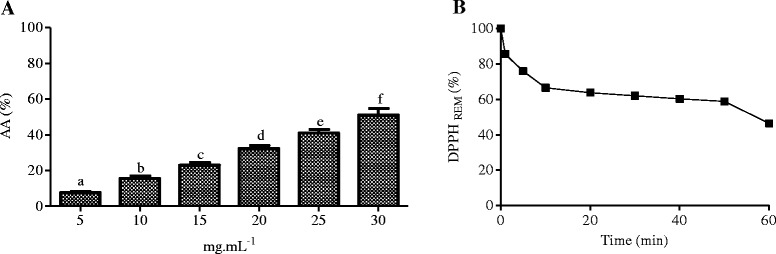
**Percentage of antioxidant activity at different concentrations of** ***B. virgilioides*** **HEE. (A).** Kinetic behavior of HEE at a concentration of 25 μg/mL to reduce DPPH free radical **(B)**. Results are expressed as the mean ± SD. The statistical difference between the concentrations was determined using one-way ANOVA, followed by Bonferroni *post-hoc* test. Different letters on the graph stand for a statistical difference between the concentrations of HEE *(p* <0.05). All experiments herein were performed in triplicate.

To verify the antioxidant potential of *B. virgilioides*, the reduction of the free radical DPPH was evaluated. HEE displayed a dose-dependent enhancement in antioxidant activity (Figure 2), with significant variations between concentrations (5–30 mg/mL), and an IC_50_ of 33.45 ± 5.97 μg/mL for 60 min.

From this data, we verified that HEE from *B. virgilioides* had an antioxidant activity index (AAI) of 0.89 ± 0.05, which classifies it as a moderate antioxidant [38]. We also observed that HEE had moderate reaction kinetics, requiring 60 min to reduce the DPPH radical level by more than half, as shown in Figure 2 via the dose–response curve, showing the percent decrease of remaining DPPH (% DPPH_REM_) over time (min).

### Redox property of *B. virgilioides* HEE

**Figure 3:**
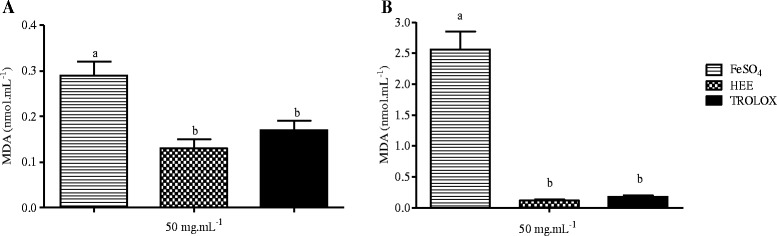
**Effect of HEE (50 μg/mL) on lipid peroxidation induced by AAPH (A) and FeSO**_**4**_ **(B).** The results are shown as the concentration of malondialdehyde formed (nmol/mL). Values are expressed as the mean ± SD. Different letters on the graph stand for statistical difference between the groups. The statistical analysis was carried out using one-way ANOVA, followed by Bonferroni *post-hoc* test (*p* <0.05). All experiments were performed in triplicate.

The hydroethanolic extract of *B. virgilioides* inhibited AAPH- and iron sulfate-induced lipid peroxidation. HEE also showed potential as a chelating agent of transition metals and neutralized Fenton reactions. HEE inhibited AAPH- and iron sulfate-induced lipid peroxidation to a similar extent (p >0.05) as the positive control, Trolox (Figure 3).

### Phytochemical profile and the total phenolic content of *B. virgilioides* HEE

**Figure 4:**
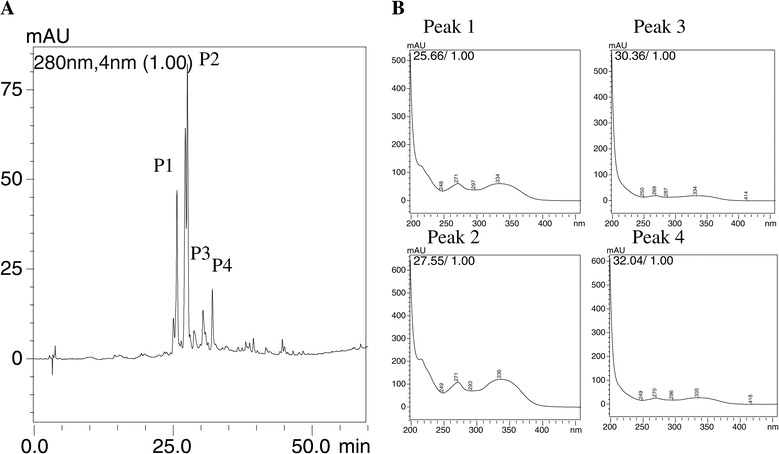
**Chromatographic profile of HEE and the respective spectra of the prominent peaks.** The experimental 5:100% water/methanol condition gradient, measured at a wavelength of 250 nm - 350 nm with the absorption spectra of UV/Vis prominent peaks **(A)** of spectra, and segmented **(B)** for each peak: Peak 1 - band “A” 271 nm, and band “B” = 334 nm; Peak 2 - band “A” 271 nm, and band “B” 336 nm; Peak 3 - band “A” 269 nm, and band “B” 334 nm; Peak 4 - band “A” 270 nm, and band “B” 335 nm.

The chromatographic profile of HEE was obtained using HPLC-DAD. The spectra showed a characteristic fingerprint of medium to high polarity substances, similar to phenolic compounds, as shown in Figure 4. These results were similar to those obtained by Im and colleagues [39].

### Quantification of the total phenol content

Total phenol content was quantified using the spectrophotometer, and determined to be 128.05 ± 26.10 mg eq AG/g *B. virgilioides* extract.

### Effect of *B. virgilioides* HEE on the reduction of oxidative stress induced by high-intensity RT

**Figure 5:**
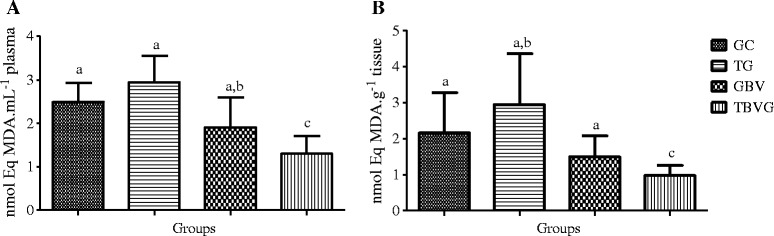
**Effect of HEE on plasma and muscular lipid peroxidation induced by high-intensity exercise. (A)** refers to plasma samples and **(B)** to muscular tissue from all animal groups: trained group (TG), trained *Bowdichia virgilioides* group (TBVG), control group (CG), and *B. virgilioides* group (BVG), each consisting of eight animals. The values represent the mean ± SD. Different letters indicate significant differences between groups (*p* <0.05). The statistical differences were determined using one-way ANOVA, followed by Bonferroni *post-hoc* test. All experiments were performed in triplicate.

To assess the effects of HEE in the body, we studied the effects of ingesting HEE in animals undergoing high-intensity RT. Oxidative stress markers were reduced in animals that ingested the *B. virgilioides*. As shown in Figure 5, we observed a significant reduction in plasma (55.68%, *p* <0.0001) and tissue (66.61%, *p* <0.0012) lipid peroxidation in TBVG rats as compared to TG rats. This finding indicates that *B. virgilioides* HEE effectively reduces oxidative stress in cellular lipid components.

**Figure 6:**
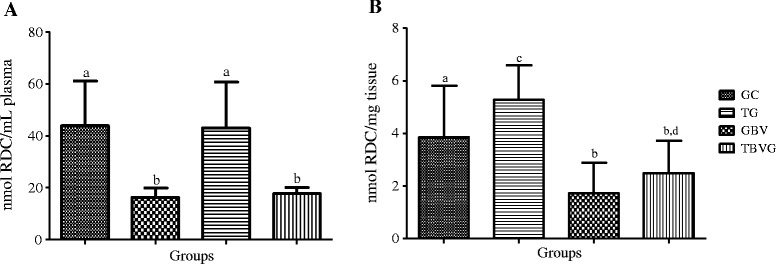
**Effect of HEE on the oxidation induced by high intensity exercise. (A)** refers to samples of plasma and **(B)** to muscular tissue from all animal groups: trained group (TG), trained *Bowdichia virgilioides* group (TBVG), Control group (CG) and Group *Bowdichia virgilioides* group (BVG), each consisting of eight animals. The values represent the mean ± standard deviation (SD). Different letters stand for significant differences between groups (*p* <0.05). The statistical differences were determined using one-way ANOVA followed by Bonferroni *post-hoc* test. All experiments were performed in triplicate.

Moreover, we also verified that *B. virgilioides* HEE efficiently prevented and/or reduced protein oxidation, as shown in Figure 6. Protein oxidation was reduced in BVG rats compared to that in CG rats, as well as in TBVG rats compared to that in TG rats. This reduction in plasma and tissue oxidation was approximately 62.83% (*p* <0.0005) and 54.97% (*p* <0.0197), respectively, in BVG and CG rats. Further, in the TBVG rats, the rate of oxidative was reduced 58.90% (*p* <0.0013) in the plasma and 52.75% (*p* <0.0059) in the muscular tissue, as compared to the TG rats.

### Effect of *B. virgilioides* HEE on the prevention of tissue damage induced by high intensity RT

The results presented in Table 2 suggest that high-intensity RT induces muscular tissue damage (Group TG *vs.* CG). There was a significant increase (173.18%, *p* <0.0001) in plasma CK in the TG rats compared to the CG rats. We also observed that the consumption of HEE during training prevented an increase in markers of tissue damage in the TBVG as compared to the TG rats, including CK, ALT, and AST.

**Table 2:** **Serum concentrations of tissue damage enzymes in** ***UI/L***

**GROUPS**	**CK ± (SD)**	**LDH ± (SD)**	**ALT ± (SD)**	**AST ± (SD)**
**CG**	198.7 ± 35.21^A^	23.61 ± 14.57^A,B^	47.15 ± 27.62^A^	128.9 ± 42.76^A^
**BVG**	199.0 ± 72.13^A, C^	8.75 ± 3.94^B^	10.05 ± 7.84^B^	92.95 ± 45.48^A^
**TG**	542.0 ± 43.00^B^	27.12 ± 17.19^A^	37.10 ± 12.57^A^	92.57 ± 23.90^A^
**TBVG**	101.4 ± 80.75^B, C^	9.25 ± 5.59^B^	9.11 ± 4.44^B^	30.03 ± 19.96^B^

CK: creatine kinase, LDH: lactate dehydrogenase, ALT: alanine aminotransferase and AST: aspartate aminotransferase. Trained group (TG), trained Bowdichia virgilioides Group (TBVG), control group (CG), and Bowdichia virgilioides group (BVG). Values with different letters stand for significant differences (p <0.05). Data presented as means plus or minus standard deviation (SD). The statistical differences were determined using one-way ANOVA followed by Bonferroni post-hoc test. (n = 8, for all animal groups).

## Discussion

In this study, we showed that intake of *B. virgilioides* HEE caused a moderate antioxidant effect *in vitro*, although a characteristic profile of substances with medium to high polarity was observed. In addition, we reported a significant reduction in markers of oxidative stress and muscle damage in resistance-trained rats treated with *B. virgilioides* HEE.

According to Wang and Huang [40], treating animals with polyphenol-based compounds can prevent lipid peroxide damage of cellular compounds. Similarly, Bansala *et al*. [41]*.* reported that products rich in polyphenols are effective in preventing both lipid peroxidation and protein oxidation in various animal tissues subjected to a high-intensity exercise protocol. This is likely due to the presence of amphipathic antioxidants, which increase their effects on cellular structures, neutralizing both intracellular and extracellular oxidizing agents [42]. Moreover, some polyphenols have significant antioxidant properties under low partial pressures of oxygen, a condition typical of skeletal muscles during intense exercise [43-45].

Phenols exhibit extensive diversity in structure and are characterized by one or more hydroxyl groups linked to an aromatic ring. They are subdivided into several categories, including simple phenols, phenolic acids (derived from benzoic and cinnamic acid), coumarins, flavonoids, stilbenes, condensed and hydrolysable tannins, lignans, and lignins, confirming the results of the phytochemical experiments [27,46]. It also confirms that these compounds are responsible for preventing lipid peroxidation, primarily due to their capacity to chelate transition metals and cellular oxidizing agents, especially those that interact with intracellular proteins [47,48].

When we assessed *B. virgilioides* HEE by using HPLC, we detected peaks with absorption spectra in UV/VIS range characteristic of phenolic compounds, including flavonoids. These absorption spectra showed variation between 250 nm and 350 nm [49]. Further, they were consistent with those reported by Im and colleagues [38], who described fingerprint characteristics of phenolic compounds of varying polarity. Data suggest that these molecules may be responsible for preventing the lipid peroxidation observed in our study. This is partly due to the ability of phenols to chelate transition metals, which inhibits cellular oxidizing agents [49]. Moreover, the compounds present in the extract are also capable of reducing lipid peroxidation, thereby neutralizing peroxyl radicals that originate from the lipid peroxidation cascade. The compounds present in *B. virgilioides* extract were also capable of reducing lipid peroxidation induced by AAPH and neutralizing peroxyl radicals, suggesting that they have an important role in the neutralization and sequestration of free radicals and chelation of transition metals, frequently acting in the initiation and propagation stages of oxidative stress [27]. This process may occur due to the phenols present in the *B. virgilioides* HEE, which were similar to those found by Dias *et al*. [50] in *Abarema cochliacarpos*, which has high antioxidant activity.

The high antioxidant activity of phenols increases with the degree of hydroxylation and depends on the rearrangement of functional groups around the nuclear structure of the molecule [51,45]. Thus, during reactions with free radicals, these compounds donate hydrogen with an unpaired electron, giving rise to another radical, which is stabilized by the rearrangement of electrons produced in the molecular resonance structure of the aromatic ring [52,53]. These studies show a significant correlation between high phenol content and antioxidant activity. Further, activity arises from the secondary metabolism in plants possessing these phenols, being primarily attributed to the hydroxyl groups attached to the aromatic ring [45,52-56]. Similar to extracts from other species, *B. virgilioides* HEE appear to have an antioxidant effect after the practice of intense RT.

RT programs such as the one adopted in this study are able to generate changes in muscle fibers owing to neural adaptations [57]. Considering that high-intensity RT causes tissue damage [42], animals subjected to the intensity of 75% of 1RM show muscular damage, as demonstrated by the increase in plasma CK compared to that in control animals that did not engage in RT. Such alterations may contribute to the development of morphological adjustments in skeletal muscles, including disruption of muscle fibers. However, one limitation of our study was the use of high-intensity RT for a period of four weeks. Thus, other studies should be made extending the period of the study.

Because high-intensity RT causes muscular damage [42], as shown by the increase in specific and non-specific markers in the serum. Numerous enzymes, such as lactate dehydrogenase from the cytoplasm of skeletal muscle fibers [58,59], AST from skeletal muscle and hepatocyte mitochondria, ALT from hepatocytes cytoplasm [60], and CK from skeletal muscles cytoplasm, increase in the serum as a result of decreased plasma membrane integrity. The animals that were treated with *B. virgilioides* HEE showed a significant reduction in all of these markers.

According to Clarkson and Hubal [61] and Deminice [19], tissue damage caused by intense exercise primarily depends on the intensity and type of exercise performed. This damage usually occurs in contractile muscle fibers and components of the cytoskeleton, causing rupture, widening, or lengthening of the Z-line, which is the contact point of contractile proteins and support the transmission of force when muscle fibers contract. Breakage of the sarcolemma may also occur [62]. The exact mechanisms involved in muscle damage induced by RT are still not fully understood [63]. However, the hypothesis that metabolic stress is associated with an increase in ROS, leading to oxidative stress, is becoming increasingly common in literature [64]. Mastaloudis *et al*. [65] stated that RT increases the metabolism of prostanoids, such as xanthine oxidase and nicotinamide adenine dinucleotide phosphate-oxidase (NADPH) oxidase, oxidation of purine bases and proteins containing iron ions, and disturbs calcium (Ca^2+^) homeostasis. These events favor increased production of oxidizing agents, triggering damage to cells and tissues [66,67]. This evidence may seem conflicting, because Kerksick and colleagues [68] reported that each marker exhibits different responses to exercise. Regarding lipid peroxidation, these authors observed no increments following an exercise program, which may be justified depending on the length, volume, and intensity of the exercise adopted in both studies. Thus, our results showed tissue damage caused by the mechanical stress of exercise, as evidenced by the increase in serum levels of the enzyme markers as well as an increase in lipid peroxidation and protein oxidation in the trained group.

This study showed that a lower degree of muscular damage and oxidative stress was observed in rats subjected to a high-intensity RT protocol after ingestion of *B. virgilioides* HEE. Some authors state that tissue damage caused by oxidative stress during high-intensity RT can be lessened through supplementation with antioxidants, such as vitamins C, E, A, and products derived from medicinal plant, including polyphenols [42,43,69,38,36].

The results of this study are in agreement with those of Panza [42], who reported that the consumption of green tea can prevent oxidative stress, as well as muscular damage in individuals engaged in high-intensity RT. Green tea is a natural product rich in polyphenols, which are excellent antioxidants capable of neutralizing the deleterious effects of free radicals and other oxidizing agents produced during physical exercise. The phenol level *B. virgilioides* HEE was moderate, and thus, showed a moderate antioxidant activity in terms of reducing DPPH free radicals.

Consuming *B. virgilioides* HEE significantly reduced lipid peroxidation in the plasma and muscles of exercised animals treated with HEE. Nevertheless, we also found that there was a reduction in the level of oxidized proteins in animals treated with HEE compared to that in those who only exercised. These data suggest that HEE can prevent or reduce muscular oxidative stress caused by high-intensity RT and minimize or prevent muscular tissue damage caused by oxidative stress. There was a reduction in plasma CK content in animals treated with HEE compared to that in the group that only engaged in exercise.

## Conclusion

Our study showed that the *B. virgilioides* HEE reduced some markers of oxidative stress and tissue damage caused by high-intensity RT for a period of four weeks. We also propose that the intake of *B. virgilioides* HEE during and/or after RT may act as an important adjuvant in the reestablishment of muscular function.
